# Brain abscess with *Ureaplasma parvum* in a patient with granulomatosis with polyangiitis

**DOI:** 10.1007/s15010-022-01966-w

**Published:** 2022-12-29

**Authors:** Marie Madlener, Marianne Breuninger, Arne Meißner, Henning Stetefeld, Sergej Telentschak, Thorsten Wille, Thilo van Eimeren, Norma Jung

**Affiliations:** 1 Department of Neurology, Faculty of Medicine, University of Cologne, University Hospital Cologne, Kerpener Straße 62, 50937 Cologne, Germany; 2 Department I of Internal Medicine, Faculty of Medicine, University of Cologne, University Hospital Cologne, Cologne, Germany; 3 Department of Hospital Hygiene and Infection Control, Faculty of Medicine, University of Cologne, University Hospital Cologne, Cologne, Germany; 4 Center for Neurosurgery, Faculty of Medicine, University of Cologne, University Hospital Cologne, Cologne, Germany; 5 Institute for Medical Microbiology, Immunology and Hygiene, Faculty of Medicine, University of Cologne, University Hospital Cologne, Cologne, Germany

**Keywords:** Granulomatosis with polyangiitis, Brain abscess, Rituximab, Ureaplasma, Case report

## Abstract

**Purpose:**

*Ureaplasma species* are associated with urogenital infections, infertility and adverse pregnancy outcomes as well as neonatal infections. Involvement of the central nervous system in adults is extremely rare. We report an unusual case of a brain abscess secondary to otitis media with *Ureaplasma parvum* in a patient with granulomatosis with polyangiitis (GPA).

**Methods:**

Imaging and laboratory findings, treatment decisions, and outcome of this case are explicated.

**Results:**

A young adult with GPA presented with progredient earache after ambulant diagnosis of otitis media. Despite different courses of broad-spectrum antibiotic therapy, she developed meningoencephalitis due to mastoiditis following temporal abscess formation. Mastoidectomy and neurosurgical abscess removal were performed. Standard cultures of cerebrospinal fluid, blood and intracranial abscess material, as well as polymerase chain reaction (PCR) for common bacterial and viral meningitis pathogens remained negative. Only eubacterial PCR of intracranial abscess material returned positive for *Ureaplasma parvum.* The patient finally improved under antibiotic therapy with moxifloxacin and doxycycline.

**Conclusion:**

*Ureaplasma species* are rare causative pathogens in immunocompromised patients. They should be considered in patients with humoral immunodeficiencies with culture-negative infections failing standard therapy. Eubacterial PCR should be performed in early states of infection in these patients for immediate diagnosis and initiation of appropriate treatment to prevent adverse outcomes.

## Case report

A 25-year-old female presented with progredient unilateral earache after ambulant diagnosis of otitis media and three days of treatment with amoxicillin (1 g three times daily). Due to a refractory status of granulomatosis with polyangiitis (GPA), she received a fourfold immunosuppression consisting of prednisolone (5 mg once daily), azathioprine (200 mg once daily), methotrexate (20 mg once weekly) and rituximab (500 mg every 6 months, last 5 months ago).

Otoscopy showed extensive inflammation of the external ear, without signs of mastoiditis. Two days after paracentesis and therapy adjustment to ampicillin/sulbactam (3 g three times daily), she presented with progredient headache, meningism and encephalopathy with aphasia.

**Fig. 1:**
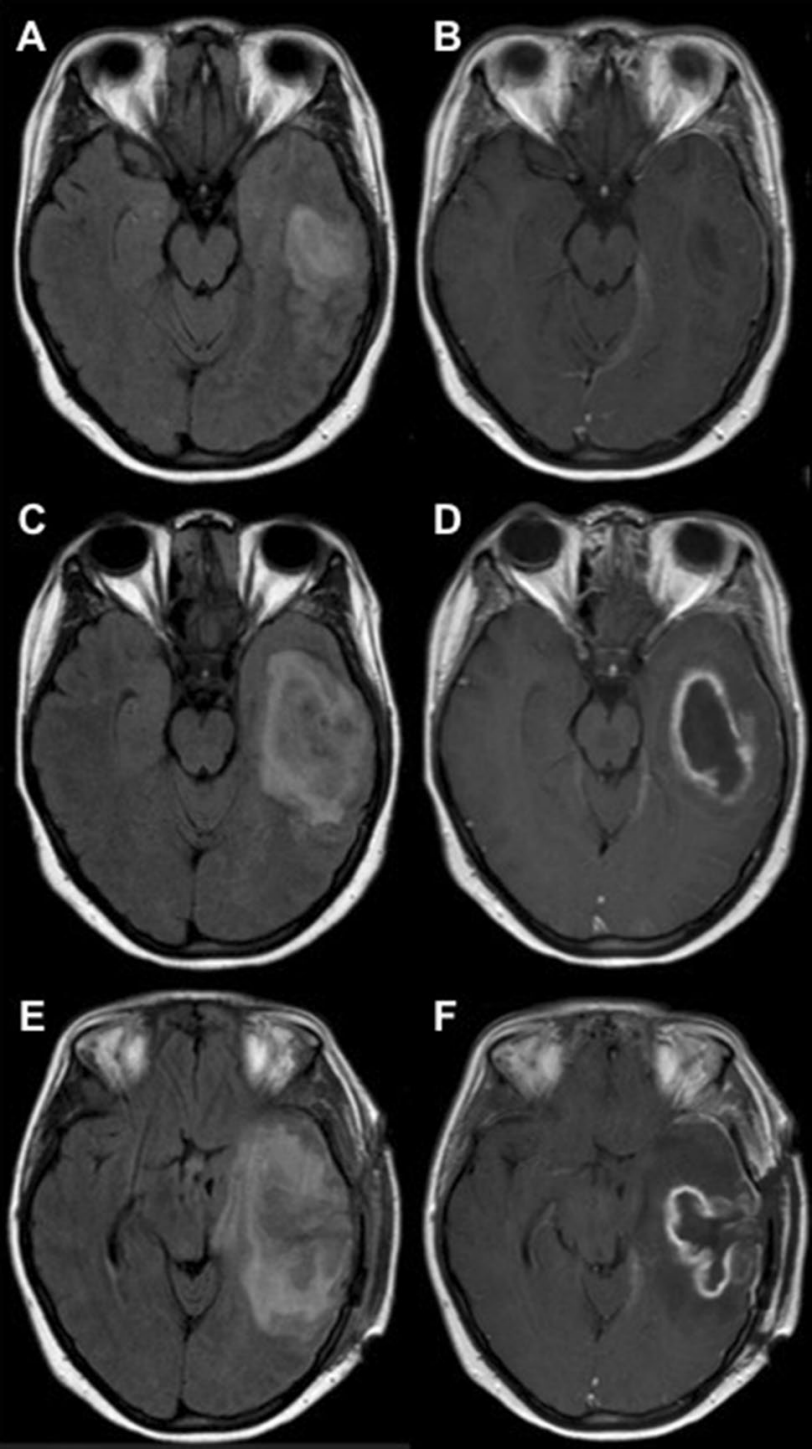
**A** Early cerebritis, axial fluid attenuated inversion recovery (FLAIR)-weighted MRI. **B** Early cerebritis, axial contrast-enhanced T1-weighted MRI. **C** Temporal abscess formation, axial fluid attenuated inversion recovery FLAIR-weighted MRI. **D** Temporal abscess formation, axial contrast-enhanced T1-weighted MRI. **E** Abscess relapse with herniation, axial FLAIR-weighted MRI. **F** Abscess relapse with herniation, axial contrast-enhanced T1-weighted MRI

Cerebrospinal fluid (CSF) assessment showed lymphocytic pleocytosis (49 white blood cells/mm^3^). Cranial computed tomography (CT) indicated mastoiditis with slight bone destruction of the temporal lobe. Treatments with ceftriaxone (2 g twice daily), ampicillin (2 g six times daily) and acyclovir (750 mg three times daily) were started for acquired meningoencephalitis due to mastoiditis. Immunotherapy was stopped and mastoidectomy was performed immediately. Cultures of CSF and blood, as well as multiplex real-time polymerase chain reaction (PCR) for common bacterial meningitis pathogens (*Haemophilus influenzae, Neisseria meningitidis, Streptococcus agalactiae, Streptococcus pneumoniae, Listeria monocytogenes* and *Escherichia coli*), enzyme-linked immunosorbent assay (ELISA) for *Borrelia burgdorferi* and *Treponema pallidum* as well as PCR for common meningitis viruses (*Cytomegalovirus, Epstein-Barr virus, Herpes simplex virus 1/2, Varicella-zoster virus* and *Enterovirus*) remained negative. Histopathology showed a florid abscessing inflammation in the mastoid and chronic granulating inflammation in the ear canal. No granulomas were found. A postoperative magnetic resonance imaging (MRI) demonstrated a temporal meningoencephalitis without abscess formation (Fig. 1). Headache and aphasia improved postoperatively.

On day 8 post mastoidectomy clinical conditions deteriorated. The patient presented with fever and reduced level of consciousness. An MRI scan identified a new temporal abscess formation, and neurosurgical abscess removal was performed. Due to insufficient improvement, antibiotic therapy was changed to ceftazidime (2 g three times daily), vancomycin (through levels of 15–20 mg/L) and metronidazole (500 mg three times daily).

Cultures of intracranial abscess material as well as PCR for *Staphylococcus aureus* remained negative, while eubacterial PCR of intracranial abscess material returned positive for *Ureaplasma parvum* (1/1 samples) on day 7 after intracranial abscess surgery (day 15 post mastoidectomy). By then, a new abscess had developed that required hemicraniectomy due to herniation. Real-time PCR confirmed *U. parvum*-DNA of secondary abscess material (2/2 samples).

Real-time PCR for *Mycoplasma* and *Ureaplasma species* confirmed *U. parvum*-DNA in 2/2 samples of secondary abscess material, while PCR for *Mycoplasma genitalum* and *hominis* as well as *Ureaplasma urealyticum* remained negative.

The patient improved under therapy with moxifloxacin (400 mg once daily) and doxycycline (200 mg loading dose, 100 mg twice daily). She was weaned from tracheostomy and percutaneous endoscopic gastrostomy. The intracranial abscess formation regressed. When transferred to rehabilitation after 10 weeks of in-patient treatment, she was fully awake and orientated, with moderate aphasia. Pr3-ANCA levels remained low, indicating a remission of GPA. Restart of immunosuppressants was deferred until significant increase of pr3ANCA levels.

No apparent signs of any genitourinary infections were apparent during the course of infection. PCR for *Mycoplasma* and *Ureaplasma species* remained negative in cervical smear. Noteworthy, urogenital examination was not performed until two weeks of calculated therapy with moxifloxacin and doxycycline.

## Discussion

We report a rare case of intracranial abscess secondary to otitis media by *U. parvum* in an adult patient with granulomatosis with polyangiitis (GPA) and fourfold immunosuppression.

*Ureaplasma species* frequently colonize the genitourinary tract of asymptomatic women. Its presence is linked to urogenital infections, infertility and adverse pregnancy outcomes as well as neonatal infections. Extragenital infections in adults have been described to cause postoperative infections or invasive infections of immunocompromised hosts, predominantly with humoral immunodeficiencies [1].

*Ureaplasma* *species* frequently colonize the genitourinary tract of asymptomatic women. Its presence has been linked to urogenital infections, infertility and adverse pregnancy outcomes as well as neonatal infections [1]. Meningitis in newborns is usually associated with the transmission of *Ureaplasma* either in utero or perinatal [2]. Extragenital infections in adults are rare. They have been described to cause postoperative infections [3–5] or invasive infections of an immunocompromised host, predominantly with humoral immunodeficiencies [6]. Another risk factor are prosthetic implants [6].

Involvement of the central nervous system is extremely rare. Only few cases in adults have been described. Previous case reports mainly described ventriculitis and meningitis [7–10]. Recent reports are connected to neurosurgical complications in immunocompetent patients [8–10]. Another case of meningitis due to *U. urealyticum* has been reported in context of immunosuppression and kidney transplantation [7]. A superinfected hematoma, which developed after the explantation of the rejected kidney graft, has been suggested as focus of hematogenous spread.

In our case intracranial *Ureaplasma* infection presented with temporal abscess formation. To the best of our knowledge, only one case with intracranial abscess formation in adults with *U. urealyticum* has been described in a patient in remission of Burkitt’s lymphoma [11]. Although the rituximab-containing treatment was administered three years ago, hypogammaglobulinemia was still present in this patient. Hypogammaglobulinemia as a risk factor for *Ureaplasma* infections was as well descried in a patient with *U. urealyticum* meningitis and Good’s syndrome [12].

The point of entry for extragenital *Ureaplasma* infections mostly remains unclear. In the previously mentioned cases of postoperative *Ureaplasma* infections a hematogenous systemic spread from the genitourinary tract after insertion of a urinary catheter was proposed. After neurosurgery a perioperative contamination is suggestive but not proofed in previous reports [8, 9]. In our case we presume infection *per continuitatem* leading to the temporal abscess formation. In line with this, MRI scans suggested primary cerebritis in direct contact to the mastoid and showed unilateral leptomeningeal contrast enhancement. Interestingly no granulomas were found in pathological work-up of the mastoid.

*Ureaplasma* are fastidious bacteria that lack a cell-wall and are therefore intrinsically resistant to antibiotics that infer with cell wall synthesis like β-lactam or glycopeptides antibiotics. They do not grow on routine media or appear on gram stain. Therefore, diagnosis is challenging. A specialized culture or PCR assay must be employed. Our case report highlights the importance of eubacterial PCR in cultures without growth. Eubacterial PCR or alternative next-generation sequencing (NGS) should be performed early in immunocompromised patients to identify rare and elusive pathogens and prevent adverse outcomes due to delayed diagnosis. Until now NGS is not routinely applied in diagnostics, but recent research highlights its potential to identify multiple pathogenic microorganisms from one sample, advantageous especially in usually polymicrobial infections like brain abscesses [13, 14].

## Conclusion

*Ureaplasma parvum* is a very rare causative pathogen responsible for invasive CNS infections in immunocompromised patients. Eubacterial PCR should be performed in early states of infection in these patients for immediate diagnosis and initiation of appropriate treatment to prevent adverse outcomes.

*Ureaplasma* *species* should be considered in patients with humoral immunodeficiencies with culture-negative infections failing standard therapy as well as in implant infections and postoperative cases.

## Data Availability

Anonymized data that support the findings of this study are available from the corresponding author upon reasonable request.
